# Effects of drought and salt stress on the root phenotype of wheat seedlings and underlying gene expression analysis

**DOI:** 10.3389/fpls.2024.1475500

**Published:** 2024-12-16

**Authors:** Kaiyue Tang, Chuanjing An, Lixia Li, Tao Sun, Jiancheng Song, Jiqiang Zhao

**Affiliations:** ^1^ School of Life Science, Yantai University, Yantai, China; ^2^ Institute of Sericulture, Chengde Medical University, Chengde, Hebei, China; ^3^ Department of Biological Science and Technology, Chengde Medical University, Chengde, Hebei, China

**Keywords:** wheat, stress, root phenotype, phytosulfokine, *TaPSK*

## Abstract

In our previous study, three *TaPSK* genes highly expressed in the roots of wheat were screened. To explore the effects of adverse stresses on the wheat root phenotype and the expression of *TaPSK3*, *TaPSK9* and *TaPSK10*, we measured the phenotypic parameters of the JM22 root system at the seedling stage after treatment with different concentrations of NaCl and PEG6000. Additionally, the relative expression levels of TaPSK3, TaPSK9, and TaPSK10 were analyzed via RT-qPCR within 72 h of treatment with 150 mM NaCl and 30% PEG6000. The results revealed that drought and salt stress significantly inhibited phenotypic parameters such as total root length, root surface area, root biomass distribution estimation and root tip number in wheat. Notably, salt stress causes wheat roots to germinate more root hairs. The expression of *TaPSK3* did not change significantly during salt stress but was upregulated approximately five-fold at 12 h of drought stress. The gene expression levels of *TaPSK9* and *TaPSK10* were upregulated to varying degrees but gradually returned to normal at 72 h. These results show that when wheat encounters stresses, the expression of *TaPSK* genes is upregulated to promote root growth and ensure the normal growth and development of plants. This study provides data and theoretical support for further study of *TaPSK* gene function and cultivation of high-quality wheat plants with strong stress resistance.

## Introduction

Environmental stresses such as high salinity and drought significantly compromise wheat survival, restrict the growth and development of wheat, and negatively impact its quality and yield. In response, plants have evolved intricate networks that perceive and respond to environmental pressures (Wang et al., 2018; Gupta et al., 2022; Wang et al., 2022). In fact, the adaptability of plants to the environment depends largely on their roots. In addition to its fixed function, it also absorbs water and various nutrients from the soil for the growth and development of the plant itself (Cavallari et al., 2021). Plants must constantly integrate root-perceived environmental signals and adjust their root structure accordingly to improve the absorption and utilization of resources. This can be achieved by enhancing the three-dimensional architecture of the root system, also known as root system architecture (RSA) (Hodge, 2009; Karlova et al., 2021; Verma et al., 2022). RSA refers to the structure and spatial modeling of roots, showing the interconnection and spatial distribution of roots of different diameter classes in soil. The plane geometry configuration mainly includes parameters such as main root length, total root length, total root tip number, total root volume, total root density, and total root surface area (Dowd et al., 2022; Duque, 2023). The three-dimensional geometric configuration is mainly reflected by the topological structure of roots, such as the occurrence of roots of different diameter classes, the three-dimensional distribution in space and the growth angle of roots (York et al., 2015; Waidmann et al., 2020). Among these, total root length refers to the overall length of the root system, reflecting the degree of development and growth of the root system; the average root diameter reflects the thickness of the root system and is related to the mechanical support strength and nutrient transport capacity. Root surface area and root volume can be used to evaluate the contact area between roots and soil, mainly reflecting the roots’ absorption capacity. Root biomass refers to the dry weight of roots per unit volume. The increase and decrease in biomass directly reflect the adaptation of organisms to the internal regulation or environmental changes (Rao et al., 2024). The above changes of RSA index have significant reference value for plant physiology-related research. For example, to withstand stress, plants may develop larger and deeper root systems (Karlova et al., 2021). Wheat root morphology and/or anatomical characteristics help the plant to sustain higher grain yields with low availability of resources, such as a relatively deeper root distribution that increases water consumption during droughts (Alrajhi et al., 2024). Therefore, understanding how wheat responds to drought and salt stress is crucial for stabilizing crop performance and preserving natural vegetation under adverse conditions (Angon et al., 2022). PSK-α is a sulfated plant peptide with the molecular formula [H-Tyr (SO_3_H) -Ile-Tyr (SO_3_H) -Thr-Gln-OH, YIYTQ] (Sauter, 2015; Hsiao and Yamada, 2020). It is derived from a precursor peptide approximately about 100 amino acids in length. After secretion, the proprotein is cleaved by proteolytic enzymes to produce bioactive PSK. YIYTQ is a highly conserved pentapeptide sequence in PSK homologs, located at the C-terminus of the precursor (Lorbiecke and Sauter, 2002; Matsubayashi, 2011; Sauter, 2015; Wu et al., 2019). PSK-α plays critical roles in various plant responses to environmental and developmental cues (Kutschmar et al., 2009). For example, studies have shown that PSK-α functions in plant cell division and differentiation, root elongation and plant resistance (Matsubayashi and Sakagami, 1996; Wu et al., 2019), the functions of PSKs have been explored various species, including *Brassica napus* (Shen et al., 2023), *Lycopersicon esculentum* (Hu et al., 2023), *Medicago truncatula* (Di et al., 2022), *Glycine max* (Yu et al., 2019), *Zea mays* (Zhang et al., 2024) and *Oryza sativa* (Yang et al., 1999; Nagar et al., 2020). In *Arabidopsis thaliana*, five *PSK* genes code for the classic YIYTQ sequence. *PSK* precursor genes are specifically expressed throughout the plant life cycle and in different cells and tissues (Lorbiecke and Sauter, 2002). indicating that PSK signal transduction occurs and functions in specific tissues.

PSK peptides interact with plasma membrane-localized transmembrane receptors (PSKRs) (Wang et al., 2015), recruiting co-receptors such as BAK1, AHAs, and cGMP-activated CNGC17. The abundance of PSKRs is tightly controlled at the post-translational level through ubiquitylation by E3 ligases PUB12/13 (Hu et al., 2023). These receptors form an integrated functional module linking Ca^2+^ influx to proton extrusion across the plasma membrane. PSK receptors are believed to have both kinase and guanylate cyclase (GC) activities (Kwezi et al., 2011; Hartmann et al., 2014; Kaufmann et al., 2017). It is inferred that CaM binding regulates the Ser/Thr kinase activity of the cytoplasmic domain of PSKRs, while the GC activity contributes to the production of cGMP (Hartmann et al., 2014; Kwezi et al., 2011). Ca^2+^ influx increases CaMs binding to auxin biosynthetic proteins YUCs, leading to auxin-mediated immunity (Zhang et al., 2018). The kinase activity of PSKRs and/or the generated Ca^2+^ signaling regulates downstream elements that confer biological functions, including cell growth and division, reproduction, legume nodulation, somatic embryogenesis, and resistance to abiotic stresses.

It was reported that PSK-α could accelerate the proliferation of meristem cells and promote the elongation of *Arabidopsis* roots (Beemster and Baskin, 1998). The overexpression of *ClPSK1* and *ClPSK2* in transgenic *Arabidopsis* also promoted the formation of adventitious roots and root elongation, with genes related to root cell growth being upregulated (Wu et al., 2019). Yang et al., isolated *Oryza sativa PSK* (*OsPSK*) cDNA encoding the PSK-α precursor in rice, which shares the characteristics of PSK precursor. It was found that *OsPSK* promoted plant cell proliferation through its product PSK-α (Yang et al., 1999). In our previous study, a total of 12 *TaPSK* genes were identified, named *TaPSK1-12*. These 12 *PSK* genes were differentially expressed at different periods and in various tissues of wheat. Additionally, bioinformatics analysis revealed that this gene family contains many cis-acting elements involved in hormone, stress, and growth responses, and it can respond to a variety of biotic and abiotic stresses, consistent with previous studies on PSK’s physiological functions. To sum up, *PSK* precursor and receptor genes are regulated by development and environmental signals at the transcription level, supporting PSK’s role in growth, development, and adaptation to biotic and abiotic stresses (Sauter, 2015). Mining the functions of *PSK* genes is of great value for root genetics research in wheat breeding. Therefore, understanding the regulation of gene expression and root phenotypes in response to salt and drought stress and their relationships can guide subsequent genetic operations.

Jimai 22 (JM22) is a medium gluten wheat variety with high yield, multi resistance and high quality. Previous studies have found that JM22 has good performance in cold resistance (Wang et al., 2023), tiller spike rate, lodging resistance and comprehensive disease resistance (Liu et al., 2021; Stührwohldt et al., 2021). It is a classic material in scientific research (Cao et al., 2024). Previously, we constructed a TILLING population containing more than 3, 200 M2 mutant lines using JM22 as the carrier, and important phenotypic separation and identification work has been carried out to the M4-M5 generations (Zhao et al., 2019). For the aboveground parts, physiological indexes, such as growth, chlorophyll content, root cell viability and chlorophyll fluorescence parameters of JM22 under different concentrations of PEG6000 drought stress and NaCl salt stress were studied. It was found that the external phenotype and internal physiology of JM22 were significantly affected by drought and salt stresses (Guo et al., 2021). However, the underground part has been less studied. This work mainly explores the phenotypes of the JM22 root system and the expression changes of related genes under drought and salt stress.

In this study, JM22 was used as the experimental material to explore the effects of NaCl and PEG6000 treatments on the root phenotypic parameters at the seedling stage and the effects of 30% PEG6000 and 150 mM NaCl treatments on the expression level of *TaPSK* genes (*TaPSK3*, *TaPSK9* and *TaPSK10*). This research provides experimental data and a theoretical basis for cultivating high-quality and high-yield wheat varieties.

## Materials and methods

### Plant materials

The wheat variety used, Jimai 22 (JM22), was provided by the Plant Stress and Molecular Breeding Base of the College of Life Sciences at Yantai University and cultivated in the greenhouse of the Molecular Biology Laboratory at Yantai University in 2023.

### Experimental design

Wheat seeds with uniform and full grains were selected and germinated in Petri dishes. On the 4th day of germination, uniformly growing JM22 plants were selected and transferred to a hydroponic tank. The samples were incubated in 1/2 Hoagland nutrient solution in a greenhouse environment for 7 days (25°C, 16 h illumination, humidity 70% ~ 75%). On the 8th day, the nutrient solution was replaced with 10%, 20%, and 30% PEG6000 or 50, 100, and 150 mM NaCl solution prepared with 1/2 Hoagland nutrient solution, and 1/2 Hoagland nutrient solution was used as the control. The data measured before treatment were recorded as 0 d, and the following root growth indices were measured at 0 d, 3 d, 6 d and 9 d: root length (cm), root volume (cm^3^), root surface area (cm^2^), root tip number (n), root average diameter (mm) and root biomass allocation estimation (mm^3^).

Transcriptome data showed that *TaPSK* gene had a relatively high transcription level during the wheat seedling period. RT-qPCR analysis was performed when the wheat growth state was stable (about two weeks). For gene expression analysis, JM22 seedlings were treated with 30% PEG6000 and 150 mM NaCl solution after two weeks. Wheat root samples were randomly taken at 0 h, 2 h, 12 h, 24 h and 72 h, and total RNA was extracted to analyze the gene expression level after stress (three replicates were set up in the experiment). The expression levels of *TaPSK3*, *TaPSK9* and *TaPSK10* were determined via RT-qPCR (Bustin et al., 2010), and the internal reference gene used was glyceraldehyde-3-phosphate dehydrogenase (*GAPDH*).

### Measurements of various root system parameters

The root data including total root length, average root diameter, total root area, total root volume, root tip count, average root diameter, root surface area, and root biomass distribution were automatically scanned with a Wanshen LA-S plant root analysis system. The treated wheat roots were washed with water, and 3 plants with uniform growth were selected for each treatment. The roots and stems of the wheat plants were separated with scissors so that the wheat plants were tiled on the measuring plate with water, and the phenotypic data of the roots were obtained via the EPSON Scan program. The above data analysis images, distribution maps, and result data can be saved and output to the Excel table.

### Quantitative real-time PCR analysis of TaPSK3, TaPSK9, and TaPSK10

Total RNA was extracted according to the instructions provided by the SPARKeasy Tissue/Cell RNA Rapid Extraction Kit (AC0202). The quality and concentration of total RNA after detection met the experimental requirements. The RNA samples were quantified and further reverse transcribed to obtain cDNA. The specific primers were used for RT-qPCR analysis, and the real-time fluorescence quantitative instrument brand model was Roche, LightCycle480II. The specific primers for fluorescence quantitative PCR were *TaPSK3* rRNA-F (5’-TAGAGGGCAAGCAGCAAGG-3’) and *TaPSK3* rRNA-R (5’-GGGTGTAGATGTAGTCGGTGTG-3’), *TaPSK9* rRNA-F (5’-CATCGGCGTGGTGAGAGTCAAG-3’) and *TaPSK9* rRNA-R (5’-CGTGTAGATGTAGTCCAGGTGCG-3’), *TaPSK10* rRNA-F (5’-CTCAAGGAGGAAGGTGGCA-3’) and *TaPSK10* rRNA-R (5’-CCCTTGTGCTGCGTGTAGA-3’), *GAPDH* rRNA-F (5’-CTGCATACGATGACATC-3’) and *GAPDH* rRNA-F (5’-TGTCACCGACAAAGTCAGTG-3’).

The amplification protocol included initial denaturation at 95°C for 3 minutes; 55 cycles of 95°C for 15 seconds, 56°C for 15 seconds, and 72°C for 5 seconds; and a final extension at 72°C for 3 minutes.

### Statistical analysis

The experimental data were first analyzed via Excel and then analyzed via GraphPad Prism v9.5 software. Before the analysis of variance, the data were tested for variance and a normal distribution, and one-way ANOVA was used to analyze significant differences of the data collected on the 9th day.

## Results

**Figure 1:**
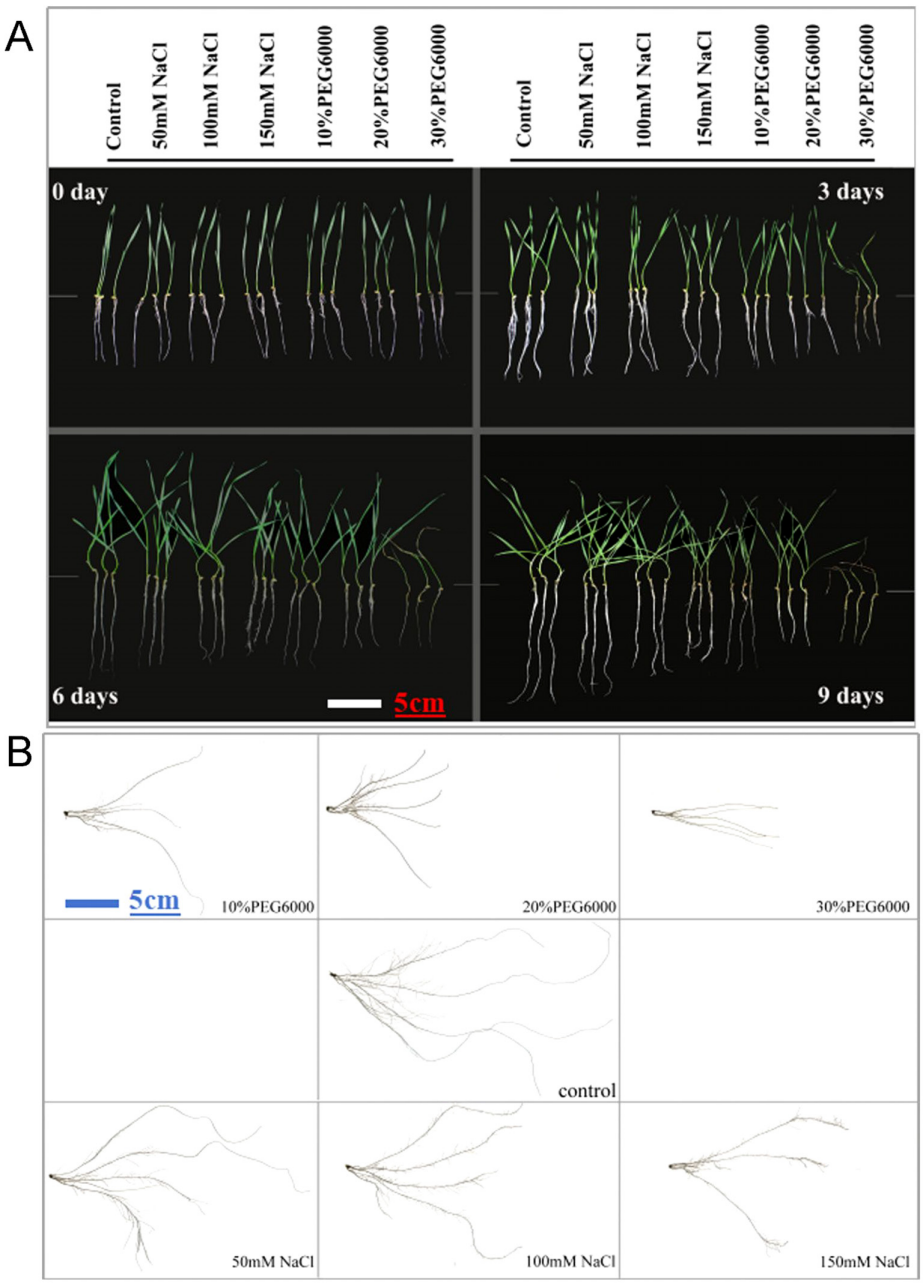
The growth and root phenotype of plants with different treatments. **(A)** Growth status of wheat at 0 d, 3 d, 6 d and 9 d under different concentrations of salt and PEG6000. **(B)** Growth of wheat treated with different concentrations of salt and PEG6000 on the 9th day. Scale bars = 5cm. In control groups, plants were cultured in 1/2 Hoagland nutrient solution without salt and PEG6000.

One-week-old wheat seedlings were subjected to stress conditions beginning on the 8th day. The root phenotypic data before treatment were recorded as those at 0 d, after which the data were measured every 3 days. **Figure 1** shows the growth phenotypes of wheat plants subjected to different concentrations of salt and PEG6000 for different durations. Treatment with different concentrations of NaCl resulted in slow plant growth, especially in terms of underground root length (**Figure 1A**). The effect of the PEG6000 treatment was more obvious than that of the NaCl treatment, especially that of the 30% PEG6000 group (**Figure 1A**). To further characterize the root phenotype, the roots of wheat plants on the 9th day under different stresses were analyzed via the Wanshen LA-S plant root analysis system. Root scanning revealed that PEG6000 treatment strongly inhibited the growth of JM22, followed by salt stress (**Figure 1B**). With increasing treatment concentration, the length of the main roots of the wheat plants clearly decreased.

Compared with those in plants treated with PEG6000, more lateral roots grew and root hairs formed in JM22 plants treated with salt (**Figure 1B**). The root shape was short and thick, whereas in the control group, only abundant lateral roots with fine and long shapes were observed in the first half of the main root (**Figure 1B**). However, when 30% PEG6000 was used to simulate drought stress, the changes in the root parameters of wheat during each time period were not obvious. On the ninth day, the outer leaves were dry, and the new leaves were still green but slightly wilted.

### Changes in the total length of the roots of wheat plants treated with different concentrations of salt and PEG6000

**Figure 2:**
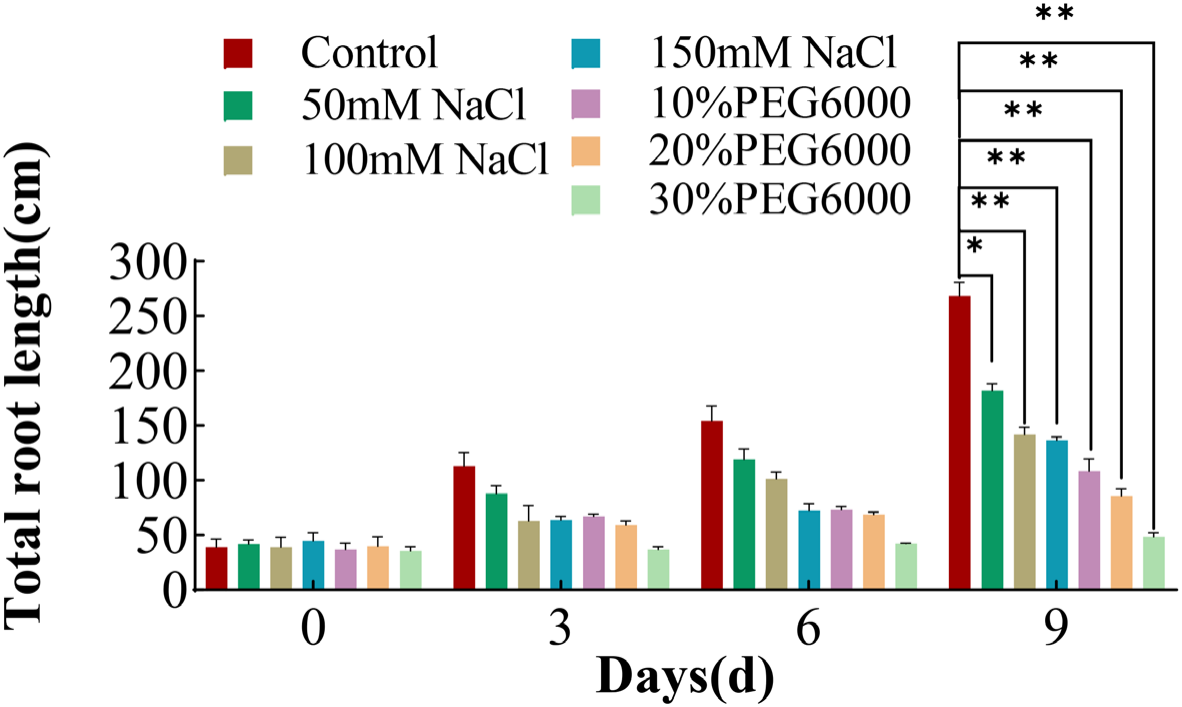
Total root length of wheat at 0 d, 3 d, 6 d and 9 d after treatment with different concentrations of NaCl or PEG6000 to simulate drought stress. Differential analysis was only carried out for the plant after treatment for 9 days. Asterisks indicate statistically significant between the control group and each stress group on the ninth day, as determined by Student’s t-test (*: p<0.05; **: p<0.01).

The total root length in both the control and treatment groups generally tended to increase with increasing stress duration, and the root growth rate of the wheat plants in the control group was greatest (**Figure 2**). The growth rate of the wheat roots was inversely related to the intensity of the applied stresses; higher stress concentrations corresponded to a slower root growth rate. The total root length of each stress group was significantly different from that of the control group. On the 9th day of stress, the total root length of the control group was 0.51~0.67 times greater than that of the different concentrations of salt stress and 0.18~0.4 times greater than that of the different concentrations of PEG6000 stress. These findings indicate that both PEG6000 and salt stress significantly inhibited the growth of wheat roots and subsequently affected the overall growth rate of the plants.

### Changes in the root surface of wheat plants under different concentrations of salt and PEG6000

**Figure 3:**
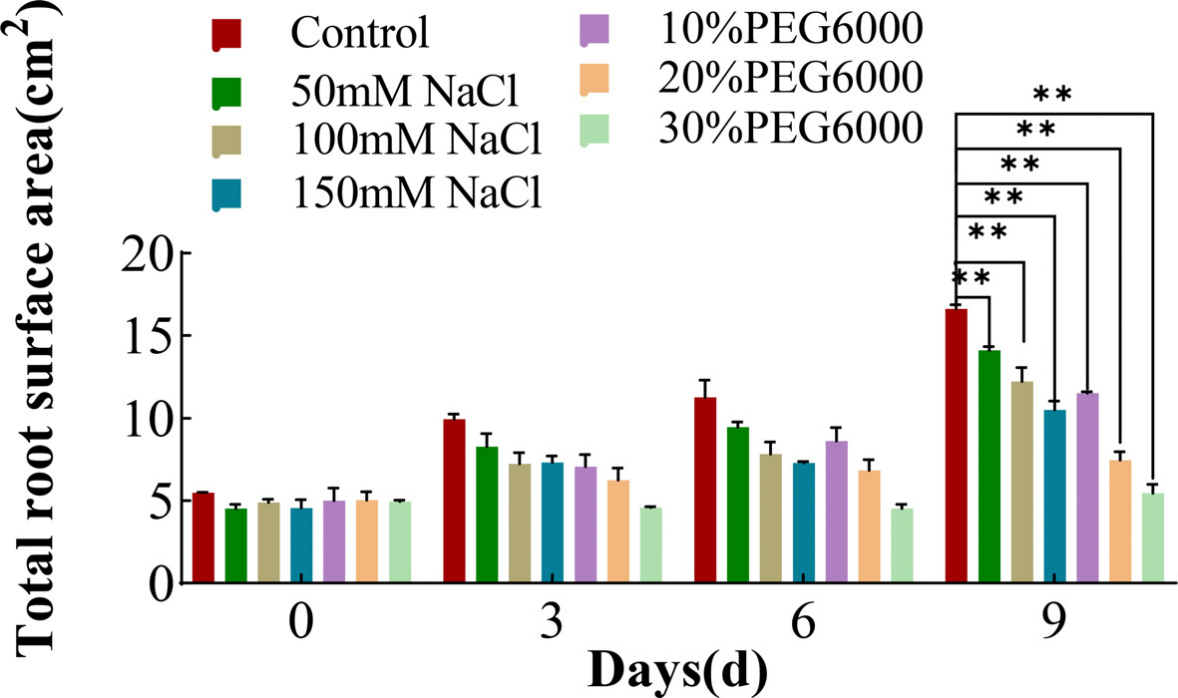
The total root surface area of wheat at 0 d, 3 d, 6 d and 9 d under different NaCl concentrations and PEG6000-simulated drought stress conditions (* *: p<0.01).

In addition to the root length, the area of the root surface was also analyzed. With increasing stress duration, the root surface area of the wheat plants in each treatment group tended to increase overall (**Figure 3**). The root surface area of the wheat plants in the control group increased the most, followed by those in the 50 mM NaCl solution treatment group. The increase in root surface area in the remaining treatment groups was relatively small, especially in the 20% and 30% PEG6000 treatment groups (**Figure 3**). On the third day, the increase in the root surface area of the plants in the 150 mM NaCl and 30% PEG6000 treatment groups was much lower than that of the plants in the control group. On the 9th day of stress, all the treatment groups presented significantly smaller root surface area than did the control group. The root surface area under different concentrations of salt stress was 0.63~0.84 times greater than that of the control group, whereas that under different concentrations of drought stress was 0.32~0.69 times greater than that of the control group. These results indicated that salt and drought stress could result in a decrease in the surface area of roots.

### Changes in the total root volume of wheat plants under different concentrations of salt and PEG6000

**Figure 4:**
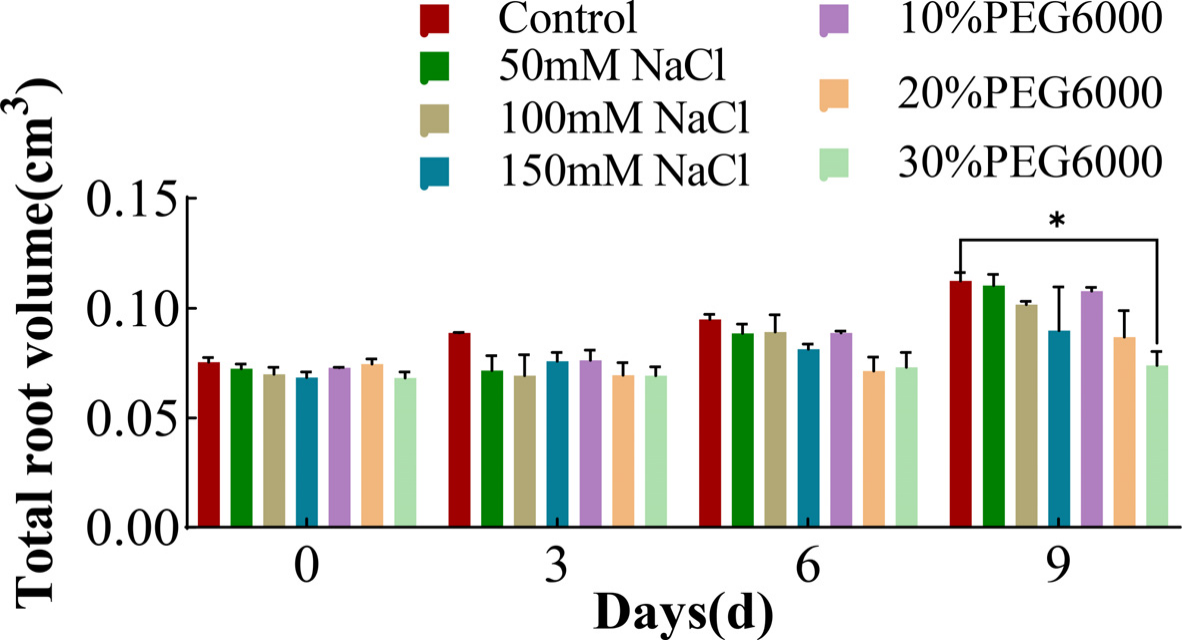
The total root volume of wheat at 0 d, 3 d, 6 d and 9 d under different concentrations of NaCl stress and PEG6000-simulated drought stress (*: p<0.05).

**Figure 4** shows the changes in the root volume of wheat in response to various concentrations of NaCl and PEG6000. As the duration of stress increased, the root volume in each group gradually increased. On the 9th day, the root volume of wheat under 30% PEG6000 drought stress was significantly smaller than that of the control group, but there was no significant difference between the other stress groups and the control group. In addition, the root volume in the 150 mM NaCl stress group was 0.2 times smaller than that in the control group. Interestingly, the total root volume in the 10% PEG6000 treatment group slightly exceeded that in the control group. These results indicated that mild drought and salt stress had little effect on wheat root volume, whereas severe NaCl stress and drought stress had inhibitory effects on wheat root volume.

### Changes in the number of total root tips in wheat plants under different concentrations of salt and PEG6000

**Figure 5:**
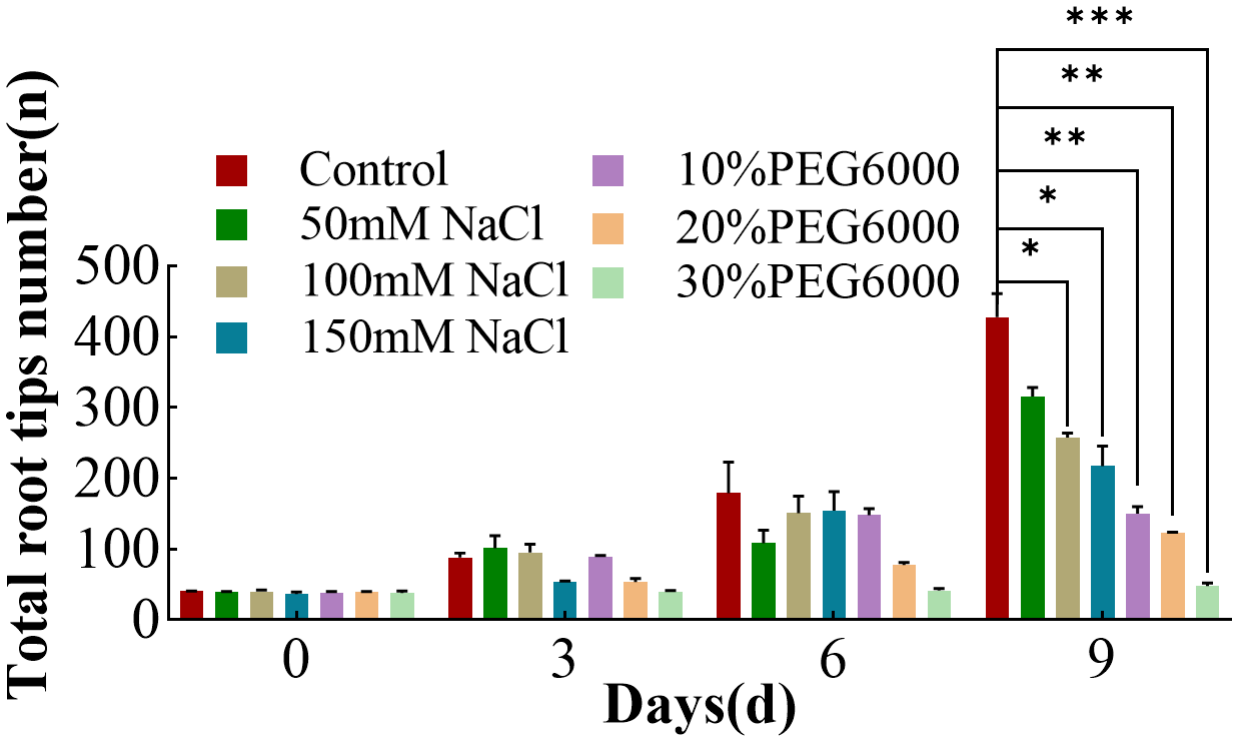
The number of total root tips of wheat at 0 d, 3 d, 6 d and 9 d under different concentrations of NaCl stress and PEG6000-simulated drought stress (*: p<0.05; **: p<0.01; ***: p<0.001).

On the 0th day of treatment, there was no significant difference in the number of root tips on the wheat plants in each group (**Figure 5**). The control group consistently presented the greatest number of root tips during the following timepoint, whereas the increase in the number of root tips in the stress treatment groups was more gradual, indicating adverse effects on root tip growth (**Figure 5**). With increasing treatment, 30% PEG6000 stress severely affected the number of root tips, and the number of root tips changed little during the growth period. Compared with that in the control group, the increase in the number of root tips in the other stress groups was relatively lower. On the 9th day, the number of root tips in the control group was 1.35~3.48 times greater than that in the other stress groups, indicating that both drought and salt stress reduced the number of wheat root tips, which was similar to previous results (Robin et al., 2021).

### Changes in the average diameter of total roots of wheat plants under different concentrations of salt and PEG6000

**Figure 6:**
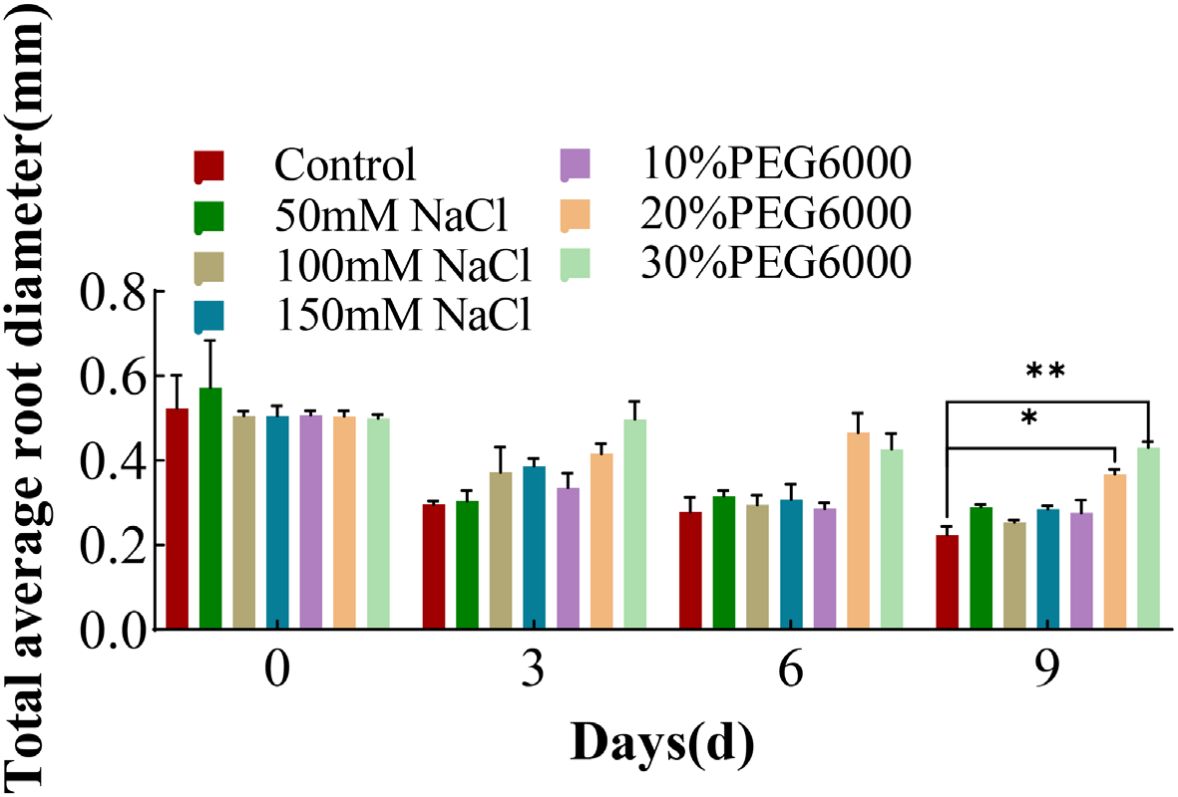
The average root diameter of total wheat roots at 0 d, 3 d, 6 d and 9 d under different concentrations of NaCl stress and PEG6000-simulated drought stress (*: p<0.05; **: p<0.01).

**Figure 6** shows the changes in the average root diameter of wheat under different concentrations of NaCl and PEG6000. The mean root diameter is the “average” diameter of all the roots of a wheat plant, including the tap roots and many lateral roots. At 0 days, there was no significant difference in the average diameter of the wheat roots among the groups. With increasing stress duration, the total root length increased, and many thin lateral roots formed. Thus, the “average” diameter of the wheat in all the groups tended to decrease. In the 6th day after treatment, there was no significant difference in the mean diameter between the treatment groups, and the average diameter of the roots in the control group was the smallest on the 9th day. The order of average root diameter by the 9th day from largest to smallest was as follows: 30% PEG6000, 20% PEG6000, 10% PEG6000, 150 mM NaCl, 50 mM NaCl, 100 mM NaCl, and the control group. The pattern is opposite to that of root growth in **Figure 2**, which shows a continuous increase in total root length for each treatment group as stress progressed.

### Evaluation of the total wheat root biomass distribution under different concentrations of salt and PEG6000

**Figure 7:**
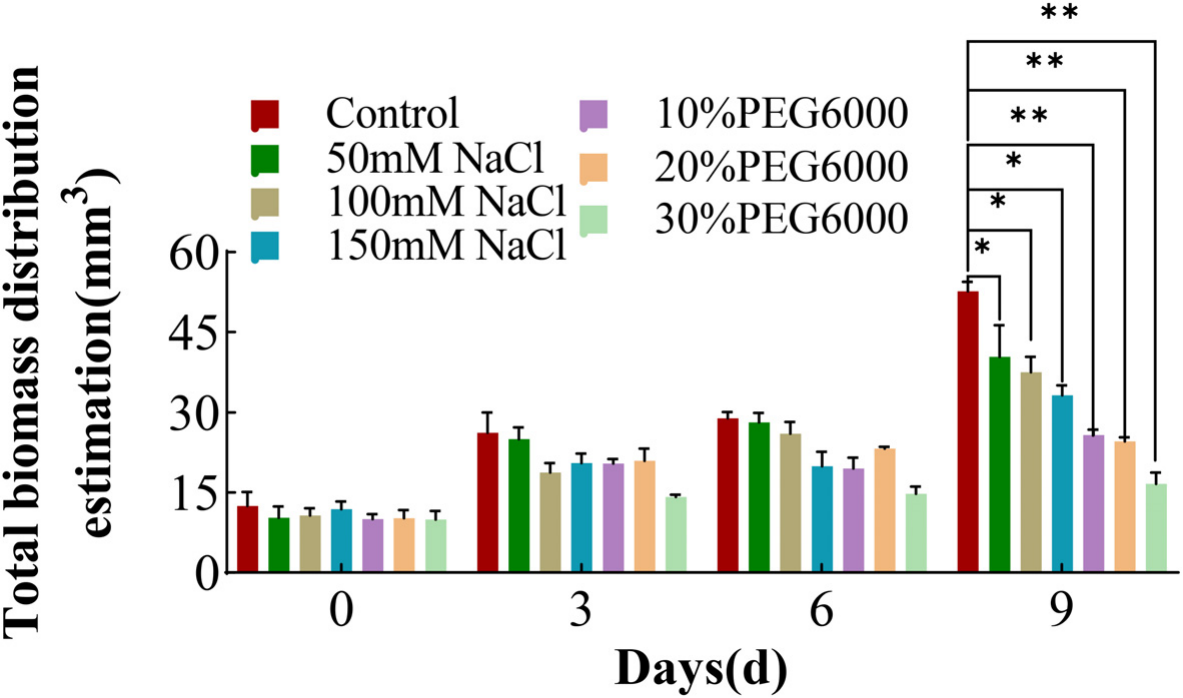
The total biomass distribution of wheat at 0 d, 3 d, 6 d and 9 d under different concentrations of NaCl stress and PEG6000-simulated drought stress (*: p<0.05; **: p<0.01).

The root biomass could reflect the overall growth status of the root or even the whole plant. Therefore, the root biomass distribution estimation from day 0 to day 9 following stress treatment was analyzed. With increasing stress duration, the biomass distribution of each treatment group also increased, and the root biomass of the control group showed greatest increase (**Figure 7**). On the 9th day of treatment, the root biomass value of the 30% PEG6000 stress group showed no significant difference from the initial measured value. The root biomass values of the other groups were consistent with the trend of the change in the total root length (**Figure 2**). These results indicated that both drought and severe salt stress significantly affected the biomass distribution of wheat roots and inhibited the growth and spatial distribution of the root structure. As the duration of stress increased, the differences in the wheat root biomass distribution became more pronounced, highlighting the substantial effects of environmental stresses on root development.

### Expression of *TaPSK3*, *TaPSK9*, and *TaPSK10*

**Figure 8:**
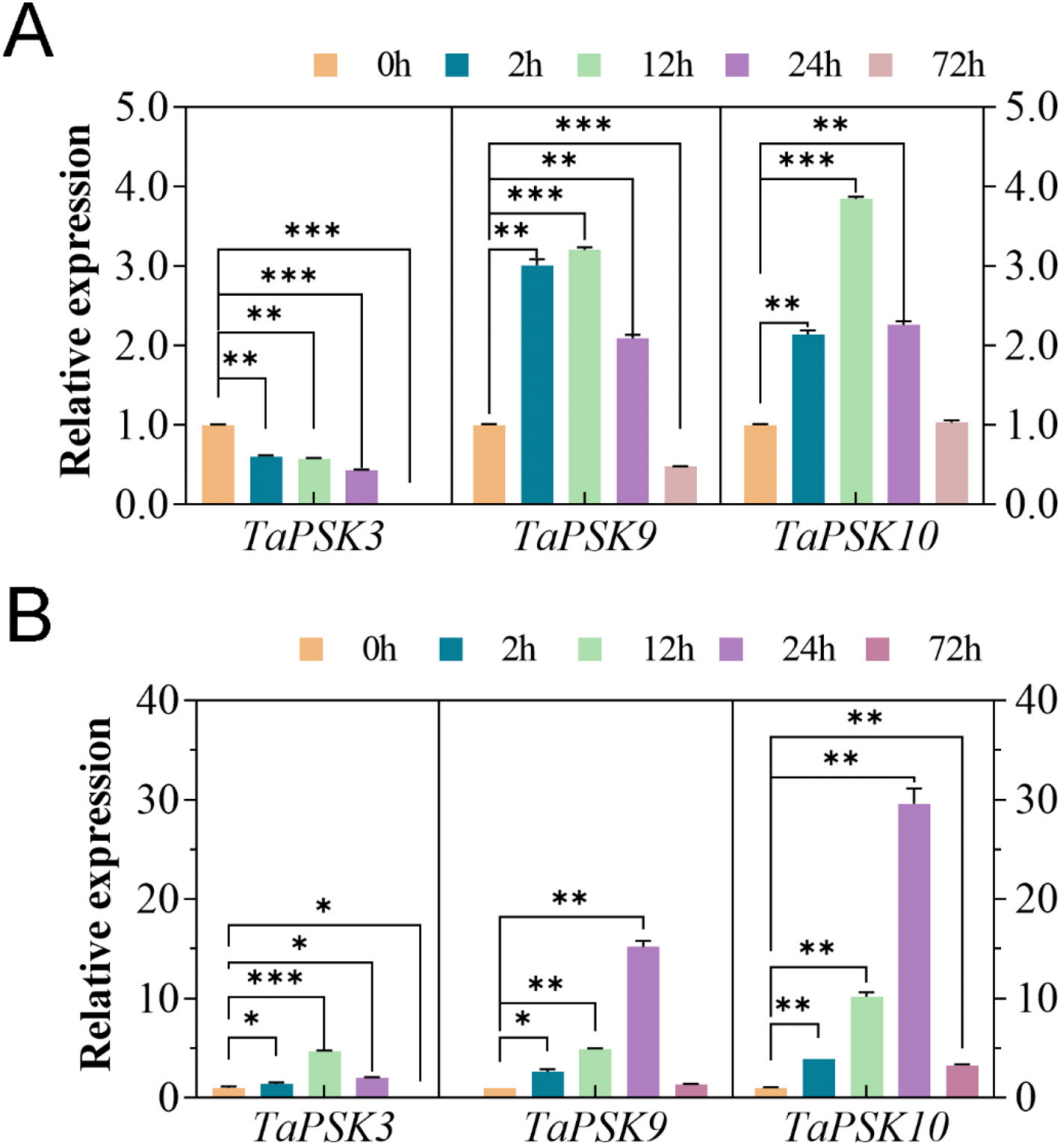
Changes in *TaPSK* gene expression in wheat plants subjected to different concentrations of NaCl **(A)** and PEG6000 **(B)**-simulated drought stress for 0 h, 2 h, 12 h, 24 h, and 72 h (**: p<0.01; ***: p<0.001). The relative expression level was normalized by comparison with that of the *GAPDH*. Various timepoints were represented by different colored histograms. The asterisk indicates that the gene expression levels of *TaPSK3*, *TaPSK9* and *TaPSK10* in wheat roots treated with 150 mM NaCl and 30% PEG6000 at 0h and other time periods. The relative expression data were expressed as mean ± SD, n = 3 biological replicates, and ANOVA test was used to obtain data with significant difference between other time periods of each gene and 0h (*: p<0.05; **: p<0.01; ***: p<0.001).

Previous studies have shown that PSKs play important roles in plant root elongation and plant resistance (Wu et al., 2019). In this study, the expression of *TaPSK3*, *TaPSK9* and *TaPSK10* in the roots of JM22 seedlings treated with 150 mM NaCl and 30% PEG6000 stress was analyzed via RT-qPCR. The relative expression levels of the three genes were normalized to 1 at 0 h. During the 72-h treatment period, the expression level of *TaPSK3* gradually decreased to the lowest level at 72 h under 150 mM NaCl stress, which was significantly lower than that of the initial measurement (p<0.01) (**Figure 8A**). In contrast, *TaPSK9* and *TaPSK10* initially increased, peaked at 12 h, and then gradually decreased. The *TaPSK10* expression level was upregulated by approximately 4-fold, and there was no significant difference between the *TaPSK10* levels at 72 h and 0 h (p>0.05). In contrast, all three genes presented upregulated transcriptional levels under 30% PEG6000 stress at 24 h (**Figure 8B**). According to the statistical results, the differences in the expression of each gene were statistically significant. Under drought stress, the gene expression of *TaPSK9* was upregulated approximately 3~10-fold and returned to the initial level at 72 h. The expression level of *TaPSK10* was upregulated by approximately 4~30-fold at different timepoints (**: p<0.01).

## Discussion

Abiotic stresses are increasingly threatening existing ecological and agricultural systems around the world. Plants respond to these stresses through sophisticated adjustments in root system architecture (RSA), involving modifications in primary root growth, branching, lateral root growth (including length and number), root hair development, and adventitious root formation, guiding root development towards more favorable conditions (Karlova et al., 2021). So, understanding root plasticity is crucial for optimizing crop responses to abiotic stresses. As we all known, plant hormones playing pivotal roles in both regulating root development and responding to abiotic stresses (Ma et al., 2024). However, the underline inter-cross talking mechanism is not well established. Phytosulfokine-α (PSKα), as a small peptide regulator, has showed significant role of in plant cell division, differentiation, root elongation, and stress resistance (Wu et al., 2019). Consequently, probing the root phenotype and expression profile of PSK genes of plants treated with salt and PEG6000 treatments will help us to uncover the potential regulation network.

Our previous studies identified 11 *TaPSK* precursor genes from wheat genome. Transcriptome data showed that these genes exhibited tissue-specific expression profile, of which *TaPSK9~10* genes were highly expressed in different root development stages, such as internode, radicle, axillary root and meristem, and especially in the root of seedlings. In addition, gene sequence analysis indicated a variety of cis-acting elements related to hormone regulation and abiotic stress response appeared in *TaPSK9~10* genes (Tang et al., 2023). This is consistent with previous studies that PSKα precursor gene plays an important role in plant root elongation and stress resistance (Wu et al., 2019). Therefore, in this study, JM22 wheat was used as experimental material to analyze the expression of *TaPSK3*, *TaPSK9* and *TaPSK10* genes under simulated drought and salt stress with PEG6000 and NaCl stresses.

Our data revealed that drought and salt stress substantially affected phenotypic traits such as total root length, root surface area, root biomass distribution, and root tip number. Under low concentration salt stress, the taproot length of JM22 seedlings decreased, but more and longer lateral roots grew. More root hairs and more lateral roots formed in the wheat plants under salt stress than in those under PEG6000 conditions (**Figure 1**). Alterations in root size, shape, and surface area, which significantly affect crop yield, are predominantly influenced by changes in root branching (Shelden and Munns, 2023; Yalamanchili et al., 2024). Our experimental observations indicated that under PEG6000 stress, the main root length of wheat seedlings decreased, and the number of root hairs decreased. Conversely, under salt stress, the roots of JM22 seedlings sprouted more root hairs and increased the number of lateral roots.

The RT-qPCR results revealed significant differences in the expression levels of *TaPSK3*, *TaPSK9*, and *TaPSK10* between drought and salt stress. The environmental stimuli led to an increase in gene expression across all the tested lines within 72 h, although the specific regulatory mechanisms varied. The genes gradually returned to normal expression levels after 72 h. Under stress conditions, lateral root growth is induced by the upregulation of lateral root induction signals to stimulate cell division (Beeckman et al., 2001), and salt stress can adapt to increase the number of roots. These findings confirm that the *TaPSK* genes increase root growth and development to cope with stress by increasing their expression levels, which provides valuable data and theoretical support for the subsequent study of *TaPSK* gene function. Comparatively, the response of the wheat *PSK* genes to drought stress was greater than that to salt stress, illustrating the differential gene expression dynamics under varying environmental pressures. This study highlights the role of the *TaPSK* genes in enhancing plant resilience against abiotic stress through upregulation, and this result can provide potential targets for genetic manipulation to improve wheat stress resistance.

Moreover, the formation and growth of lateral roots, which are regulated by various hormones, are key factors in controlling total RSA (Liu et al., 2003; Márquez et al., 2019; Zhang, et al., 2022b). Under salt stress, gene expression and hormonal regulation in wheat seedlings are significantly altered. Salt stress primarily disrupts osmotic pressure and ionic balance, triggering complex gene regulatory networks and hormone signaling pathways. Studies have shown that genes related to salt tolerance, such as antioxidant enzyme genes (e.g., *SOD*, *CAT*, *POD*) and osmoprotectant-related genes (e.g., *P5CS1*, *BADH*), are notably upregulated in wheat seedlings under salt stress, helping to mitigate oxidative damage and maintain osmotic balance. In terms of hormonal regulation, abscisic acid (ABA) plays a crucial role in the salt stress response by regulating genes involved in stomatal closure to reduce water loss. ABA also modulates downstream stress-responsive genes, including transcription factors like DREB, WRKY and MYB. Additionally, levels of ethylene, auxin (IAA), and cytokinins fluctuate under salt stress, influencing root growth, cell division, and overall stress adaptation (Zhu, 2002). PEG6000 is commonly used to simulate drought stress, and under PEG treatment, gene expression and hormonal regulation in wheat seedlings undergo significant changes. PEG6000 induces osmotic stress by lowering the water potential in plant cells, activating drought-responsive mechanisms. Genes such as dehydration-responsive element-binding proteins (DREB) and aquaporins (PIP) are significantly upregulated under PEG treatment, helping to mitigate water loss and improve drought tolerance (Shinozaki and Yamaguchi-Shinozaki, 2007). Regarding hormonal changes, abscisic acid (ABA) is rapidly accumulated in response to PEG-induced osmotic stress, triggering the expression of drought-responsive genes. Furthermore, levels of auxin (IAA) and gibberellins (GA) generally decrease under drought conditions, inhibiting excessive growth and ensuring the allocation of energy to essential stress response pathways (Zhang et al., 2022a).

Based on *PSK* gene expression results, *PSK* genes are highly expressed under both salt stress and PEG6000 treatment, although their expression patterns are different. Considering the role of PSK peptides in root growth, we speculate that PSK might be part of the IAA hormonal regulatory network and positioned relatively downstream of IAA regulation. In Wu et al. ‘s study, up-regulation of *PSK* gene expression can promote the expression of genes related to root development, such as *PLT1*, *PLT2*, *SHR*, *SCR* and *WOX5* (Wu et al., 2019), Through this, PSK regulate downstream gene expression through PSKR, thereby participating in the regulation of root growth and development. Given that *PSK* genes contain binding sites for hormone-regulated transcription factors, our next step will be to identify the transcription factors involved in regulating PSK gene expression, as well as to study *PSK* gene mutants. This will play a crucial role in further understanding the underlying mechanisms.

## Data Availability

The original contributions presented in the study are included in the article/supplementary material. Further inquiries can be directed to the corresponding author.
